# Structure–Activity Analysis and Molecular Docking Studies of Coumarins from *Toddalia asiatica* as Multifunctional Agents for Alzheimer’s Disease

**DOI:** 10.3390/biomedicines8050107

**Published:** 2020-05-02

**Authors:** Pitchayakarn Takomthong, Pornthip Waiwut, Chavi Yenjai, Bungon Sripanidkulchai, Prasert Reubroycharoen, Ren Lai, Peter Kamau, Chantana Boonyarat

**Affiliations:** 1 Faculty of Pharmaceutical Sciences, Khon Kaen University, Khon Kaen 40002, Thailand; ppitcha.t@gmail.com (P.T.); bungorn@kku.ac.th (B.S.); 2 Faculty of Pharmaceutical Sciences, Ubon Ratchathani University, Ubon Ratchathani 34190, Thailand; pwaiwut79@yahoo.com; 3 Faculty of Sciences, Khon Kaen University, Khon Kaen 40002, Thailand; chayen@kku.ac.th; 4 Center for Research and Development of Herbal Health Products, Khon Kaen University, Khon Kaen 40002, Thailand; 5 Department of Chemical Technology, Faculty of science, Chulalongkorn University, Bangkok 10330, Thailand; Prasert.R@chula.ac.th; 6 Kunming Institute of Zoology, the Chinese Academy of Sciences, Kunming 650223, China; rlai@mail.kiz.ac.cn (R.L.); kmpeter26@gmail.com (P.K.)

**Keywords:** acetylcholinesterase, amyloid beta aggregation, neuroprotection, molecular docking, multi-target drug, structure–activity relationship

## Abstract

Coumarins, naturally occurring phytochemicals, display a wide spectrum of biological activities by acting on multiple targets. Herein, nine coumarins from the root of *Toddalia asiatica* were evaluated for activities related to pathogenesis of Alzheimer’s disease (AD). They were examined for acetylcholinesterase (AChE) and AChE- or self-induced amyloid beta (Aβ) aggregation inhibitory activities, as well as neuroprotection against H_2_O_2_- and Aβ_1–42_-induced human neuroblastoma SH-SY5Y cell damage. Moreover, in order to understand the mechanism, the binding interactions between coumarins and their targets: (i) AChE and (ii) Aβ_1–42_ peptide were investigated *in silico*. All coumarins exhibited mild to moderate AChE and self-induced Aβ aggregation inhibitory actions. In addition, the coumarins substituted with the long alkyl chain at position 6 or 8 illustrated ability to inhibit AChE-induced Aβ aggregation, resulting from their dual binding site at catalytic anionic site and peripheral active site in AChE. Moreover, the most potent multifunctional coumarin, phellopterin, could attenuate neuronal cell damage induced by H_2_O_2_ and Aβ_1–42_ toxicity. Conclusively, seven out of nine coumarins were identified as multifunctional agents inhibiting the pathogenesis of AD. The structure–activity relationship information obtained might be applied for further optimization of coumarins into a useful drug which may combat AD.

## 1. Introduction

Alzheimer’s disease (AD), the most common cause of dementia, is a chronic and progressive neurodegenerative disorder. The main clinical manifestation of AD is memory impairment, leading to the progressive loss of attention, emotions, mental capacity, and the ability to learn [1,2]. The molecular mechanisms of AD are still unclear; however, several studies explained that AD is related to cholinergic deficiency [3], amyloid beta peptide (Aβ) aggregation [4], hyperphosphorylation of tau protein [5], and oxidative stress [6].

Several studies suggested that memory impairment results from a decline of acetylcholine (ACh), which is the neurotransmitter that plays roles in the encoding of new memories and information [7]. Therefore, AD treatments are currently based on the inhibition of acetylcholinesterase (AChE). AChE, which locates at post-synaptic membranes, is an enzyme that hydrolyses the ester bond of acetylcholine (ACh) into acetate and choline, leading to termination of the action of ACh [8]. AChE is composed of three binding sites including the catalytic site (CAS), anionic site (AS), and peripheral anionic site (PAS). The CAS has a catalytic triad composed of three principle amino acids including Ser 200, Glu 327, and His 440, which are directly involved in ACh hydrolysis. The AS is located in the middle gorge and consists of Trp84, Tyr130, and Phe330. The PAS at the entry to the gorge is represented by Tyr 70, Asp72, Tyr121, Tyr 334, and Trp279 [8,9]. Interestingly, several studies suggested that AChE was not only involved in the hydrolysis of ACh, but also accelerated the aggregation of Aβ into amyloid fibrils [10,11,12]. Moreover, researchers indicated that AChE interacts with Aβ and induces Aβ fibril formation at the PAS of AChE. Aβ interacts with the hydrophobic region of AChE to stabilize the AChE–Aβ complex [10]. Therefore, the dual binding agents that interact simultaneously with both the CAS and PAS sites of AChE might exhibit dual inhibition of AChE and Aβ-aggregation.

Aβ aggregation also plays a key role in AD progression [13]. The Aβ peptides in the brain are generated from the amyloid precursor protein (APP) through sequential cleavages by β- and γ-secretase enzyme activities. Aβ is accumulated in the brain and spontaneously aggregates into the oligomers to form insoluble fibrils that turn to amyloid plaques, which initiate mitochondrial oxidative stress and promote hyperphosphorylation of tau proteins [14], resulting in neurotoxicity. 

Currently, there are only five drugs approved by the Food and Drug Administration (FDA) for AD treatment. However, the current approved drugs that modulate one target could only enable a palliative treatment instead of curing AD. Due to the multi-pathogenesis of AD, the classical single-target approach may be inadequate. Therefore, searching for candidates that target at multiple sites of the pathologic cascade has become an effective strategy for developing drugs against AD [15,16,17]. Natural products from plants have been considered as an important medical reservoir holding enormous potential. The natural products possessing multifunction characteristics were found to have a potential for the treatment of AD such as galantamine isolated from *Galanthus woronowii* Losinsk and Huperzine A obtained from *Huperzia serrata* [18,19]. Thus, the natural compounds acting against a wider range of targets associated with pathologic cascade might afford additional benefits for AD therapy. 

**Figure 1:**
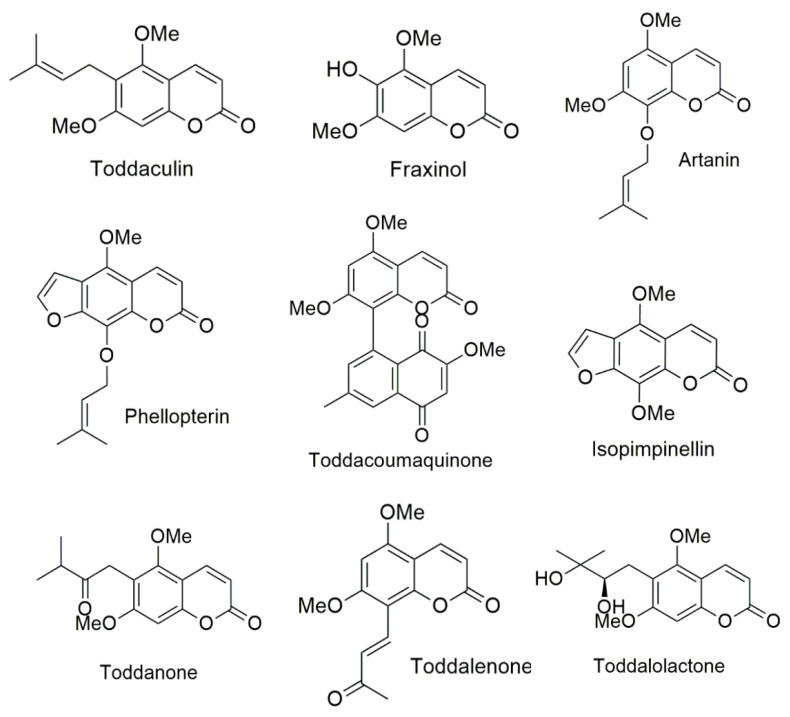
Structures of coumarin derivatives.

In the present study, we focused on *Toddalia asiatica* (L.) Lam, which is a medicinal plant of the family Rutaceae. This plant is widely distributed in many countries of Africa and Asia [20]. The plant is used traditionally to treat several diseases including malaria, stomachache, and chest pains [21,22]. Coumarins are the major components found in *T. asiatica*. Coumarins have been studied for various pharmacological effects, such as anti-inflammatory [23], anti-cancer [24], anti-oxidant [25], anti-coagulant [26], and anti-microbial [27] activities, as well as anti-AChE activity [28,29]. They display a wide spectrum of biological activities by acting on multiple targets. Thus, in this study, we focused on the coumarins that can interact with AChE, both of PAS and CAS, to discover the novel therapeutic agents that can increase the amount and duration of acetylcholine (ACh) action, as well as prevent Aβ aggregation. Nine coumarins extracted from the root of *T. asiatica* include phellopterin, isoimpinellin, toddalolactone, toddaculin, toddacoumaquinone, toddalenone, toddanone, artanin, and fraxinol (Figure 1). They were evaluated for biological activities related to AD, including AChE inhibitory and AChE- and self-induced amyloid beta (Aβ) aggregation inhibitory activities. In order to understand the mechanism, the binding interactions between coumarins and their targets AChE and Aβ peptide were investigated *in silico* by molecular docking. Moreover, the most potent multifunctional coumarin was evaluated for neuroprotective effects against H_2_O_2_- and Aβ-induced cell damage.

## 2. Materials and Methods

### 2.1. Coumarin Derivatives

Nine coumarins extracted from the root of *Toddalia asiatica*, including phellopterin, isopimpinellin, toddalolactone, toddaculin, toddacoumaquinone, toddalenone, toddanone, artanin, and fraxinol (Figure 1), were gifted by Chavi Yenjai (Faculty of science, Khon Kaen university, Thailand). The isolation and structural elucidation were explained in a previous report [30,31]. Acetylthiocholine iodide (ATCI), bovine serum albumin (BSA), beta amyloid 1-42 (Aβ1–42), tacrine, trolox, N-acetyl cysteine (NAC), trypsine, fetal bovine serum (FBS), and Dulbecco’s modified Eagle medium nutrient mixture F-12 (DMEM/F12) were purchased from Sigma-Aldrich (SM Chemical supplies Co., Ltd., Bangkok, Thailand), Merck (Merck, Bangkok, Thailand), Gibthai (GT Chemical supplies Co., Ltd., Bangkok, Thailand), and Fluka (SM Chemical supplies Co., Ltd., Bangkok, Thailand). AutoDock program version 4.2.6 (the Scripps Research Institute, La Jolla, CA, USA) and BIOVIA Discovery Studio 2017 (BIOVIA, San Diego, CA, USA) were used in the molecular docking study.

### 2.2. Acetylcholinesterase Inhibitory Activity

Acetylcholinesterase inhibitory activity was determined and modified from Ellman, as described elsewhere [32]. Briefly, the assay mixture consisted of 25 µL of 0.1 M phosphate buffer (pH 7.4), 25 µL of 1 mM substrate (acetylthiocholine iodide solution), 125 µL of 1 mM 5, 5′-dithiobis-(2 nitrobenzoic acid (DTNB), 50 µL of 0.2 Units/mL AChE from *Electrophorus electricus* (type VI-S), and 25 µL of test inhibitors in various concentrations (0–100 μM). The stock solutions of test inhibitors were dissolved in DMSO and diluted with phosphate buffer so that the final concentration of DMSO did not exceed 0.1%. The enzyme activity was measured by the increase in absorbance at 405 nm for 5 min with a METERTECH Accureader M965 microplate reader. The enzyme activity and the percentage inhibition were determined. The compound concentration producing 50% of AChE inhibition (IC_50_) was calculated from a concentration-inhibition curve using linear regression analysis. All determinations were carried out at least three times and in triplicate wells.

### 2.3. Inhibition of AChE-Induced Aβ_1–42_ Aggregation

Inhibition of AChE-induced Aβ_1–42_ aggregation was modified and measured using a thioflavin T (ThT) binding assay, as described elsewhere [33,34]. Briefly, Aβ_1–42_ was dissolved in 50 mM phosphate buffer (pH 8.0). Three µL of Aβ_1–42_ (250 µM) and 10 µL of AChE (10 unit/mL) or PBS in the presence and absence of 2 µL of test inhibitors (0–500 μM) were co-incubated for 3 h at 37 °C. To quantify amyloid fibril formation, the mixture was diluted to a final volume of 200 µL with 5 µM ThT in glycine–NaOH buffer (pH 8.5) after the incubation period, and the fluorescence measurement was obtained at the excitation and emission wavelengths of 446 nm and 490 nm, respectively. The fluorescence intensities were recorded, and the percentage of inhibition on aggregation was calculated by using the following equation: (1 – IFi/IFc) * 100%, in which IFi and IFc were the fluorescence of the AChE-induced Aβ_1–42_ aggregation group in the presence and absence of the test compounds, respectively, after subtracting the fluorescence of self Aβ_1–42_ aggregation and the background fluorescence of 5 μM ThT in the blank buffers. The compound concentration producing 50% of inhibition of AChE-induced Aβ_1–42_ aggregation (IC_50_) was calculated from a concentration-inhibition curve by using linear regression analysis. All determinations were carried out at least three times and in triplicate wells. Curcumin was used as a reference compound.

### 2.4. Inhibition of Self-Induced Aβ_1–42_ Aggregation

Inhibition of self-induced Aβ_1–42_ aggregation was assayed, as described elsewhere [34]. Briefly, nine microliters of 25 μM of Aβ_1–42_ in 50 mM phosphate buffer, pH 7.4, was incubated with 1 μL of the test compound at various concentrations (0–500 μM) in the dark at 37 °C for 48 h. After incubation, the samples were mixed with 50 μM glycine/NaOH buffer (pH 8.5) containing 5 μM ThT. Fluorescence intensities were measured at an excitation wavelength of 446 nm and an emission wavelength of 490 nm. The fluorescence intensities were recorded, and the percentage of inhibition on aggregation was calculated by using the following equation: (1 − IFi/IFc) * 100% in which IFi and IFc were the fluorescence intensities obtained for Aβ_1-42_ aggregation group in the presence and absence of the test compounds, respectively, after subtracting the background fluorescence of 5 μM ThT in the blank buffers. The compound concentration producing 50% of Aβ_1–42_ aggregation inhibition (IC_50_) was calculated from a concentration-inhibition curve for each compound using linear regression analysis. The experiment was done in independent triplicates.

### 2.5. Computational Studies

The target templates AChE and Aβ_1–42_ were constructed by removing water and other solvent molecules, adding all hydrogens, assigning Gasteiger charges, and merging non-polar hydrogen atoms. The AChE template was prepared from a crystal structure of *Tc*AChE bound with the AChE inhibitor, N-[8-(1,2,3,4-tetrahydroacridin-9-ylthio)octyl]-1,2,3,4-tetrahydro acridin-9-amine, 2CEK and validated by crystallographic structures of six AChE inhibitors (2CEK, 1H22, 1ZGB, 2CMF, 1ODC, and 1UT6). The Aβ fibril template was prepared using the X-ray crystal PDB code 2BEG. AutoGrid was used to generate grid maps. The grids were designated to include the active site of AChE and the whole fibril structue. The grid box dimensions were defined with a size of 80 × 70 × 70 and 120 × 60 × 40 Å for AChE and Aβ fibrils, respectively, and the grid spacing was set to 0.375 Å. All ligands were docked by using the Lamarckian genetic algorithm via the AutoDock 4.2.6 program. The Lamarckian genetic algorithm protocol was set using a population size of 100 individuals with 100 ligand orientation runs. Additionally, the energy evaluation was 1,000,000 and the maximum number of evaluation was 27,000. The orientation with the lowest docked energy was considered as the best conformation. After the docking process, the docking complex poses were analyzed for their interactions by using BIOVIA Discovery Studio 2017.

### 2.6. Neuroprotective Activity against Hydrogen Peroxide (H_2_O_2_) and Aβ_1–42_ Toxicity

Human neuroblastoma cells (SH-SY5Y) were cultured in Dulbecco’s Modified Eagle Medium (DMEM/Ha’s F12) containing 50 IU/mL penicillin, 50 g/mL streptomycin, 2 mM l-glutamine, and 10% fetal bovine serum. Cells were maintained at 37 °C in a humidified incubator in an atmosphere of 5% CO_2_ For the assay, the SH-SY5Y cells, at a density of 5 × 10^5^ cell/mL, were seeded in a 96-well plate and incubated for 48 h.

For cytotoxicity evaluation, the cells were treated with potential coumarin derivatives in various concentrations for 24 h. After that, cell viability was determined by staining the cells with 3-(4, 5-dimethyl-2-thiazolyl)-2, 5-diphenyl-2H-tetrazolium bromide, and MTT (5 mg/mL in PBS) for 2 h. The optical density of each well was measured at 550 nm in a METERTECH Accureader M965 microplate reader.

For neuroprotection against H_2_O_2_ investigation, the cells were pretreated with coumarin in various concentrations for 24 h. After removing unabsorbed test compounds by washing with phosphate buffer saline, the cells were treated with 100 µM H_2_O_2_ for 2 h to induce oxidative stress. Cell viability was determined by the MTT colorimetric method. The optical density of each well was measured at 550 nm. Curcumin at the concentration of 10 µM was used as a reference standard. All data were expressed as a percentage of non-H_2_O_2_-treated groups (control group). The cell viability of the control group was expressed as 100%. The experiment was done in independent triplicates (4 wells/group).

For neuroprotection against Aβ_1–42_ toxicity, the Aβ_1–42_ preparation was modified from [17]. Briefly, lyophilized Aβ_1–42_ was reconstituted in sterile water and keep at −80 °C until further use. Aliquots were diluted with a culture medium to achieve a final concentration of 25 μM and then incubated at 37 °C for 72 h to form aggregated amyloid. For the assay, SH-SY5Y cells were incubated with aggregated Aβ (25 µM), with or without the test compounds, for 24 h. Cell viability was determined by the MTT colorimetric method. The optical density of each well was measured at 550 nm in a METERTECH Accureader M965 microplate reader. Curcumin (10 µM) was used as a reference standard. All data were expressed as a percentage of non-Aβ_1–42_-treated groups (control group). Cell viability of the control group was expressed as 100%. The experiment was done in independent triplicates (4 wells/group).

The results are expressed as mean ± SD (*n* = 3). Statistical significance was determined by paired *t*-test (two group comparison). For all statistical analysis, significance levels were set at *p* value < 0.05.

## 3. Results and Discussion

### 3.1. Acetylcholinesterase Inhibition

The inhibitory activity of coumarin derivatives against AChE was evaluated according to the spectrophotometric method of Ellman using tacrine, an AChE inhibitor, as a positive control. The ability of the test compounds to inhibit AChE function was shown as IC_50_, which is the test compound concentration that resulted in 50% inhibition of AChE activity. The AChE inhibitory activity of coumarins is summarized in Table 1. All coumarins exhibited moderate inhibitory activity with IC_50_ values in the range of 17 to 53 µM. To elucidate the correlation between the inhibitory activity and the substitution groups on the coumarin ring, all compounds were divided into three groups according to substitution on position 6, substitution of furan ring, and substitution on position 8. As indicated in Table 1, fraxinol, which possesses a small hydroxyl group at position 6 of the coumarin ring, showed the highest inhibitory potency toward AChE. It was more potent than toddanone, toddaculin, and toddalolactone, which bear a longer side chain. The replacement of hydroxyl group with long alkyl chain might decrease electrostatic distribution in coumarin ring and might generate the steric hindrance that decrease the binding affinity between coumarin and AChE, resulting in diminished inhibitory activity [16]. The presence of a furan ring at position 6–7 showed an improved inhibitory activity. Apparently, phellopterin (IC_50_ = 38 μM) was more potent than artanin (IC_50_ = 51 μM), which does not bear any furan ring in the coumarin ring. Similarly, the presence of furano- or pyranocoumarins slightly increased the AChE inhibitory activity in natural coumarins [35], which could indicate that the furan ring possessing high electron density might influence the increase in binding affinity with AChE. Our results revealed that phellopterin, which possesses a longer chain substituent at position 8 of the coumarin moiety, showed similar effect with isopimpinellin, which contains a methoxyl group. However, some studies reported that the inhibitory activity of the natural coumarins can be increased when presented with larger substituents at position 8 [35,36]. Our present study showed that the coumarin moiety is the essential part of AChE activity. The presence of bulkier substituents at position 6 influenced a decrease in the activity, which was also found in [15]. Moreover, we suggested that substitution of the furan ring at position 6–7 might improve AChE inhibitory activity by increasing the binding affinity.

### 3.2. AChE-Induced Aβ Aggregation Inhibitory Activity

Several pieces of evidence indicated an association between AChE and Aβ. Apart from enhancing hydrolysis of ACh, AChE also plays a role in Aβ aggregation promotion by forming a complex with Aβ at the hydrophobic region of PAS [12,37]. To explore the effects of nine coumarins on AChE-induced Aβ aggregation inhibitory activity, a ThT-based fluorescence assay was performed, and curcumin was used as a positive control. The inhibitory effects of the test compounds are summarized in Table 1. Seven of nine coumarins showed an ability to inhibit Aβ_1–42_-aggregation induced by AChE. Isopimpinellin and fraxinol did not have inhibitory effects. Among the tested inhibitors, toddacoumaquinone exhibited the most potent activity with IC_50_ value of 72 μM. The substitution at position 6 of the coumarin ring played a vital role, for instance, fraxinol, which bears a small hydroxyl group, did not have an inhibitory effect against AChE-induced Aβ aggregation, while coumarin, which has a longer side chain like todaculin, toddalolactone, and toddanone was active. In addition, the presence of di-hydroxyl or carbonyl groups on the long alkyl chain at position 6, similar to toddalolactone (logP = 1.84, calculated by Swiss ADME program) and toddanone (logP = 2.61), resulted in enhanced activity, comparable to toddaculin (logP = 3.34). The inhibitory activity seemed to relate with hydrophilicity of the substituents at position 6. The presence of a furan ring in the coumarin ring did not affect Aβ aggregation induced by AChE. Apparently, phellopterin and artanin possessed similar inhibitory activities toward the AChE-induced aggregation of Aβ_1–42_. For position 8, substitution with a bulky group enhanced the inhibitory effect against Aβ aggregation induced by AChE. The lipophilicity of the substitution group at position 8 seems to play a role in the inhibitory action. The results showed that toddacoumaquinone (logP = 3.33) possessed better activity than artanin (logP = 3.03) and toddalactone (logP = 3.34), respectively.

### 3.3. Self-Induced Aβ Aggregation Inhibitory Activity

To determine the inhibition of self-induced Aβ_1–42_ aggregation of coumarins, we used a ThT fluorescence assay to monitor and quantify aggregation of Aβ, and curcumin was used as the reference compound. All coumarins exhibited inhibitory effects with IC_50_ values ranging from 76 to 220 µM (Table 1). Toddanone, phellopterin and todalenone showed the most potent with IC_50_ less than 100 μM. Structure–activity relationship analysis indicated that the lipophilicity has impacts on inhibitory activity. Coumarins which possessed log P less than 2, similar to fraxinol (logP = 1.51) and toddalolactone (logP = 1.84), provided unfavorable effect, which displayed inhibitory activity with IC_50_ values higher than 200 μM. However, the balance between hydrophilic and lipophilic properties should be concerned. We found that the substitution with higher lipophilic group at position 6 or 8 led to declined inhibitory potency; for instances, compared among the coumarins bearing different substituents at position 8, toddalenone (log P = 2.30) showed higher inhibitory activity than artanin (log P = 3.03) and toddacoumaquinone (log P = 3.3), respectively. Our results also demonstrated that the furan substitution of phellopterin (IC_50_ = 95 μM) at position 6–7 exhibited higher inhibitory activity than the corresponding OCH_3_-substituted artanin (IC_50_ = 124 μM). The furan ring might increase the binding affinity by electron delocalization.

### 3.4. Binding Interaction Study by Molecular Docking

#### 3.4.1. Binding Interaction with AChE

**Figure 2:**
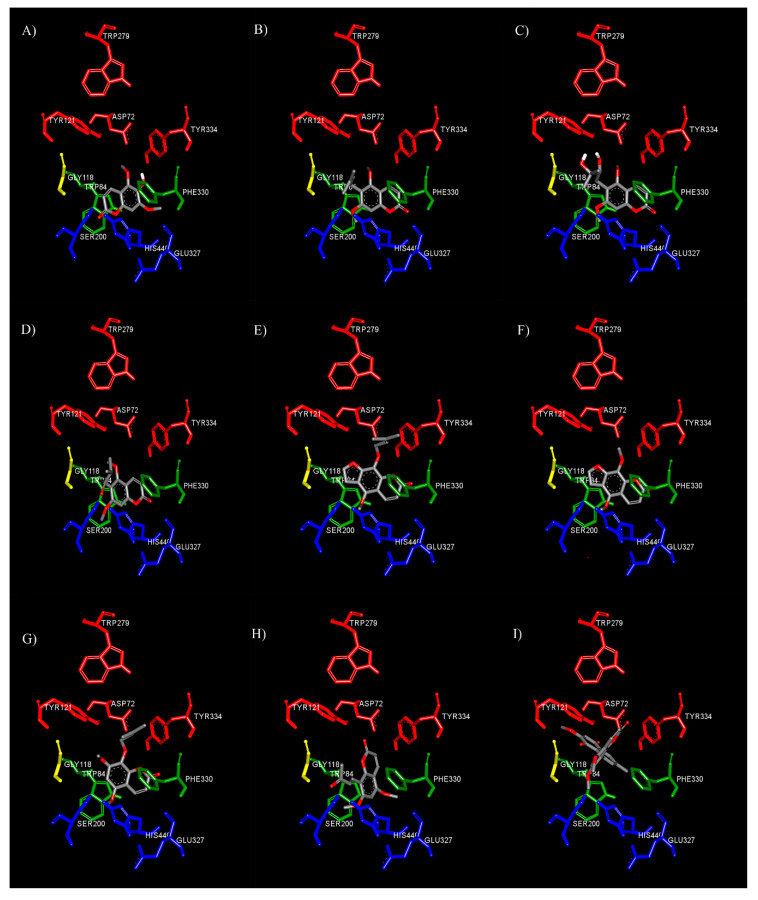
Binding mode of coumarin derivatives fraxinol (**A**), toddaculin (**B**), toddalolactone (**C**), toddanone (**D**), phellopterin (**E**), isopimpinellin (**F**), artanin (**G**), toddalenone (**H**), and toddacoumaquinone (**I**) at the binding site of AChE predicted by molecular docking. The active sites are coded with different colors (CAS; blue, AS; green, oxyanion hole; yellow, and PAS; red).

To clearly understand the mechanism of action between our test compounds and AChE, a molecular modeling technique was performed to determine the binding orientation. The binding orientations of nine coumarins in AChE are shown in Figure 2. The docking results revealed that the coumarin ring of all derivatives was bound to the catalytic anionic site (CAS) of AChE. The coumarin moiety had a planar structure, which is inserted into the CAS of AChE. Therefore, our results suggested that the coumarin moiety is the important structure for AChE inhibitory action by interfering with ACh hydrolysis at the CAS binding site. Some studies showed that the coumarin moiety binds preferably to interact at the PAS of AChE, and they are mostly modified the long chain substituents at positions 3 or 4 of coumarin ring [12,37], but Fellarero et al. [29] demonstrated that coumarin could interact with Ser200 at CAS and have π–π stacking interaction with Phe330. Thus, our data seem to support that substitutions of the alkyl chain at position 6 or 8 forced coumarin ring locating at CAS. Therefore, the coumarin moiety could interact with both the CAS and PAS of AChE depending on the substituents. 

In the case of toddalenone (Figure 2H) and toddacoumaquinone (Figure 2I), their binding orientations were quite different from other coumarins. In the middle gorge, the coumarin ring of toddalenone had π–π stacking and π–sigma interactions against Phe330 and Trp432, respectively, and the methoxy group at position 5 formed hydrogen bonds with Tyr442 and Trp84. Moreover, the carbonyl group on alkyl chain at position 8 could also form hydrogen bonds with Ser200, Gly118, and Gly119. Similarly, toddacoumaquinone could form π–π stacking interactions with Phe330 and Tyr121. Interestingly, the naphthoquinone ring, which was substituted on position 8, could interact with Tyr121 and the indole ring of Trp84 to form hydrogen bonds and π–π interactions, respectively. Moreover, the methyl group on the naphthoquinone ring also formed two π–sigma interactions with Tyr334 and Trp432 in the PAS. The result indicated that the naphthoquinone ring at position 8 located in PAS binding site. 

With regard to the substituent at the 6^th^ position of the coumarin ring, substitution of bulky groups or long side chains affected AChE inhibitory activity. The docking study showed that fraxinol (4.28 Å, a distance between oxygen atom at position 1 on coumarin ring and carboxylic group of His440) could enter the active site CAS closer than toddaculin (4.90 Å), toddanone (5.22 Å), or toddalolactone (4.92 Å), which had bulky substitution groups (Figure 2A). The substitution of bulky moieties might generate the steric effect and related to the decrease of binding affinity between coumarins and CAS. Then, this might cause the decrease of AChE inhibitory activity. Interestingly, the long alkyl chain at position 6 could interact with Tyr121, which is a key residue of PAS. The alkyl side chain of toddaculin (Figure 2B) and toddanone (Figure 2D) established π–alkyl interaction with Tyr121, while toddalolactone (Figure 2C) interacted with Tyr121 via hydrogen bonds. Therefore, these compounds exhibited a multi-target profile encompassing inhibitory activities toward AChE and Aβ aggregation induced by AChE, resulting from their dual site to CAS and PAS, as predicted by the docking study.

Additionally, the influence of a furan ring and the length of side chain at position 8 on the inhibitory potency was examined with phellopterin (Figure 2E), artanin (Figure 2G), and isopimpinellin (Figure 2F). The addition of a furan ring of phellopterin compared to artanin revealed that the furan ring seemed to increase the affinity because it could form π–π stacking against the indole ring of Trp84. Moreover, the longer alkyl chain established π–alkyl interactions with peripheral anionic site residues, including Tyr121, Phe330, and Tyr334. Likewise, the alkyl chain of artanin formed π–alkyl interactions with Tyr121, Phe330, and Tyr334. 

Thus, our data suggested that coumarins exhibited the inhibitory activity of AChE by binding to CAS and AS. Moreover, the presence of long alkyl side chains at positions 6 or 8 allowed for additional interaction with the PAS, thereby enabling a dual-site interaction. Previous studies revealed that AChE could induce Aβ fibril formation by forming AChE–Aβ complexes throughout the PAS of AChE [12,37]. Our docking results were also relevant to our AChE-induced Aβ aggregation inhibitory activity results. The test inhibitors with the alkyl chain interacting with amino acid residues of the PAS exhibited the inhibition of AChE-induced Aβ aggregation. Therefore, these data supported that coumarins with the long alkyl chain were able to enhance cholinergic tone by reducing the hydrolytic activity of AChE and prevent the deposition of Aβ fibrils as dual action inhibitors.

#### 3.4.2. Binding Interaction with Aβ

In order to get further insight into the binding mode, we performed a docking study to generate the binding mode for coumarins on Aβ_1–42_ (Protein Data Bank (PDB) ID: 2BEG). The assemblies of Aβ are composed of monomers, soluble oligomers, and insoluble fibrils. The soluble oligomers have different sizes which are ranging from dimers, trimers, tetramers, pentamers, and dodecamers [38]. The folded monomers are self-associates to form nucleus for fibril elongation, which derived a paranucleus [39]. After that, the paranuclei self-associate to form protofibrils and sequentially become fibrils. The Aβ monomers could bind with the hydrophobic stretch of residues 17–21 at the tip of the protofibrils [40], which Ile41 was essential for mediating paranucleus formation and Ala42 was required for self-association of paranuclei [41]. Based on previous studies [36,41,42], Aβ_1–42_ fibrils have parallel and in-register β-sheets, of which they have inter-sheet interactions to stabilize conformation between two β-strands. The two strands composed of residues 12–24 and 30–42 are connected by a turn at residue 25–29. In addition, to stabilize the structure and connect the two β strands, there are inter-sheet contacts which are formed by hydrophobic interactions between Phe19 and Gly38, and Ala21 and Val36, as well as a salt bridge between Asp23 and Lys28.

**Figure 3:**
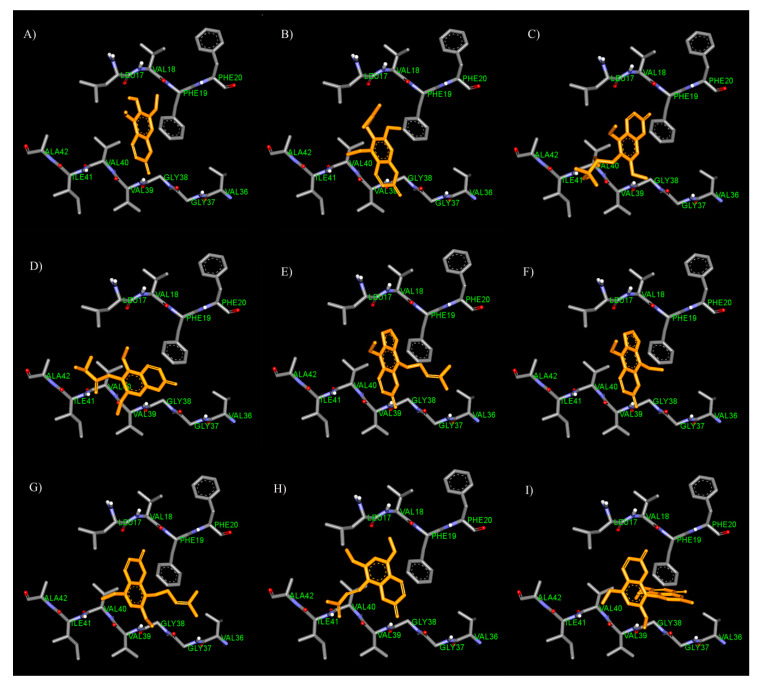
Binding mode for interactions of fraxinol (**A**), toddaculin (**B**), toddalolactone (**C**), toddanone (**D**), phellopterin (**E**), isopimpinellin (**F**), artanin (**G**), toddalenone (**H**), and toddacoumaquinone (**I**) docked into Aβ_1–42_ fibrils.

Considering the results, the coumarin ring of all test inhibitors could interact with Aβ_1–42_ at the inter-sheet against Phe19 and Val40 via π–π and π–sigma interactions, respectively (Figure 3). According to the significance role of Phe19 on amyloid aggregation, Cukalevasi et al. [43] reported that the substitution of Phe19 with leucine dramatically has a slower aggregation rate than the wild type. Moreover, Phe19 also formed the inter-sheet contact to stabilize the Aβ structure as above. Therefore, these data supported that the coumarin ring could interfere with Aβ destabilization through the π–π interaction with Phe19. In the case of fraxinol (Figure 3A), it was found that its hydroxyl group at position 6 and carbonyl group at position 2 could form hydrogen bonds with Leu17 and Val39, respectively. With regard to the substituents at the 6^th^ position of the coumarin ring, the docking results revealed that the alkyl chain with two hydroxyl groups at position 6 of toddalolactone (Figure 3C) could bind to the residue Val39 with hydrogen bonds. However, the carbonyl group on alkyl chain of toddanone (Figure 3D) and the vinyl group on alkyl chain of toddaculin (Figure 3B) could form a hydrogen bond and a van der Waals interaction, respectively, with Ile41 which it is the crucial residue for promoting the initial oligomerization of Aβ_42_ [41]. Therefore, they possessed a higher activity than fraxinol and toddalolactone.

For the comparison between the furan ring and methoxy group at position 6–7, the results showed that phellopterin with furan ring (Figure 3E) exhibited better activity than artanin, which contains a methoxy group (Figure 3G). The docking results indicated that the furan ring which has high electron delocalization could form π–π interaction with the aromatic ring of Phe19 and Val40. The furan ring of isopimpinellin (Figure 3F) is also stacked against the aromatic ring of Phe19 through the π–π interaction. The results suggested that the addition of the furan ring seemed to afford the binding affinity between the coumarins and Aβ.

Additionally, the substitution at position 8 showed that the length of the substituents affected the inhibitory activity. Compared to isopimpinellin, phellopterin bearing longer alkyl chain at position 8 possessed higher activity than isopimpinellin. The docking result showed that the long alkyl chain at position 8 of phellopterin is expanded to bind with residues Ala21 and Val36 via π–alkyl interactions (Figure 3E). Similarly, the long alkyl chain of artanin (Figure 3G) could interact with residues Ala21 and Val36, which could indicate that the presence of long alkyl side chain at position 8 allowed to gain additional interaction with Aβ, thereby increasing the binding affinity.

### 3.5. Neuroprotective Activity against H_2_O_2_ and Aβ_1–42_-Induced Neuronal Cell Death

**Figure 4:**
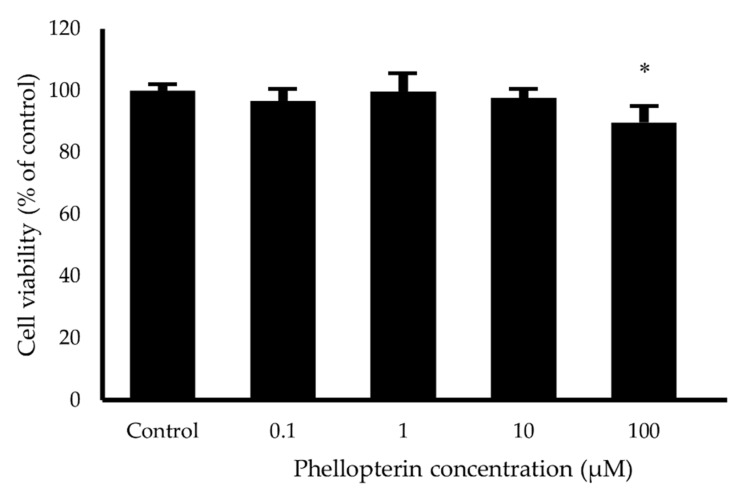
Effects of phellopterin on cell viability of SH-SY5Y cells. The values are reported as mean ± SD (*n* = 4), Paired t-test was used to compare between control and test inhibitors, * *p* < 0.05.

For neuroprotective evaluation, we selected phellopterin as the most potent compound which possessed the multimode-action of anti-AChE, AChE- and self-induced Aβ aggregation inhibition. To evaluate the cytotoxicity of phellopterin in human neuroblastoma SH-SY5Y cells, the MTT assays revealed that there was no significant change in the viability of SH-SY5Y cells after treatment with the phellopterin at concentration of 0.1 to 10 µM for 24 h (Figure 4). Thus, this data indicates that phellopterin at those concentrations are non-toxic for use in further neuroprotective effects investigations.

Oxidative stress has been widely implicated in neuronal cell death, which has been considered as the pathogenic mechanism underlying neurodegenerative disorders including AD [44]. Oxidative stress is characterized by the imbalance in the production of reactive oxygen species (ROS) and antioxidative defense systems which are responsible for the removal of ROS. ROS are able to initiate cell death. H_2_O_2_ is commonly used as an exogenous source of ROS and neuronal cells exposed to H_2_O_2_ undergo cell death with oxidative stress [45]. Several studies reported that H_2_O_2_ could induce oxidative stress in neuronal cell by several pathways such as free radical generation, oxidative enzyme induction, intracellular signaling pathway activation, etc [46,47,48,49]. Therefore, an H_2_O_2_-induced cytotoxicity model is considered suitable for the study of neurodegeneration induced by oxidative stress.

**Figure 5:**
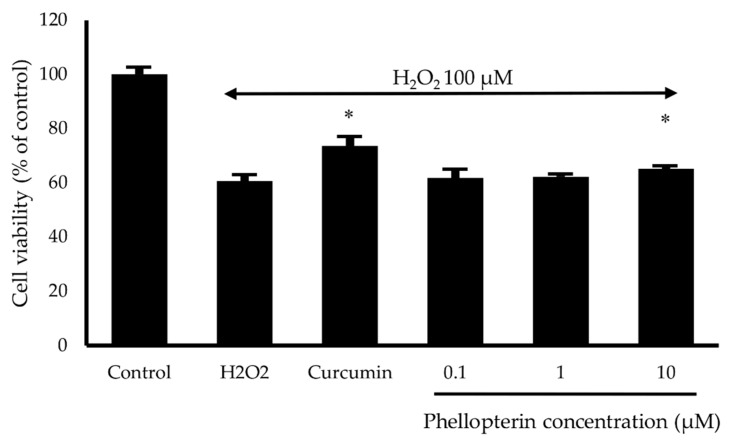
Effects of phellopterin on H_2_O_2_-induced cell damage in SH-SY5Y cells. Curcumin at 10 μM was used as a reference standard. The values are reported as mean ± SD (*n* = 3). Paired t-test, * *p* < 0.05 versus H_2_O_2_ treated group.

The neuroprotective effect of phellopterin against oxidative stress was determined by colorimetric MTT assay in SH-SY5Y cells. Hydrogen peroxide at concentration 100 µM was used to induce oxidative damage. SH-SY5Y cells were pretreated with phellopterin at a concentration of 0.1 to 10 µM for 24 h prior to exposure to H_2_O_2_ for 2 h. The H_2_O_2_ treatment decreased the cell viability to 60.61%; however, pre-incubation with 10 µM of phellopterin significantly attenuated the H_2_O_2_-induced cell damage compared to the H_2_O_2_ treated group (Figure 5). Thus, these findings indicated that phellopterin exhibited potential protective effects against oxidative stress induced by H_2_O_2_ in SH-SY5Y cells.

**Figure 6:**
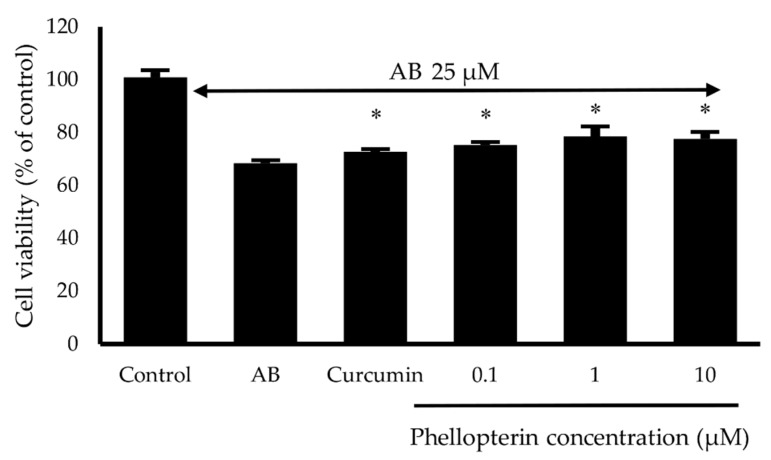
Effects of phellopterin at the concentration of 0.1 to 10 μM on Aβ_1–42_-induced cell damage in SH-SY5Y cells. Curcumin at 10 μM was used as a reference standard. The values are reported as mean ± SD (*n* = 3). Paired t-test, * *p* < 0.05 compared to Aβ_1–42_ treated group.

Aβ has been implicated in the pathogenesis of AD. Several evidences demonstrated that Aβ protein-induced neurotoxicity is a major pathological mechanism of AD and leads to neuronal cell death [13,50,51]. Therefore, the neuroprotective effect against Aβ_1–42_ toxicity of phellopterin was also determined in SH-SY5Y cells. Aβ_1–42_ at concentration 25 µM was treated with cells in the presence or absence of phellopterin at a concentration 0.1 to 10 µM for 24 h, and then the cell viability was determined by MTT assay. Our results showed that phellopterin could significantly increase cell viability compared to only the Aβ_1–42_-induced group in SH-SY5Y, as shown in Figure 6. Several studies indicated that Aβ could induce neuronal cell damage by evoking a cascade of oxidative damage to neurons [52,53]. The neuroprotective effect of the coumarins might partly come from anti-oxidative stress action. Taken together, these findings suggested that phellopterin significantly protected H_2_O_2_ and Aβ-induced cell damage in SH-SY5Y cells.

## 4. Conclusions

In summary, nine coumarins from *Toddalia asiatica* root were evaluated for activities related to the pathogenesis of Alzheimer’s disease, including anti-AChE function, AChE- and self-induced Aβ aggregation. Seven out of nine coumarins were identified as multifunctional compounds inhibiting the pathogenesis of AD. The most potent multifunctional coumarin, phellopterin showed an ability to protect neuronal cell damage induced by H_2_O_2_ and Aβ_1–42_. The structure–activity relationship and molecular docking studies suggested that the substituents on position 6, 7, and 8 of coumarin ring influenced anti-AChE function and anti-Aβ aggregation. The structure–activity relationship information obtained from this study might be useful in the development of new anti-AD drugs.

## Figures and Tables

**Table 1:** Biological activities of coumarins related to Alzheimer’s disease (AD).

Compound	AChE assay IC_50_ (μM)	AChE-Induce Aβ IC_50_ (μM)	Self-Induced Aβ IC_50_ (μM)
Phellopterin	38 ± 3	97 ± 7	95 ± 3
Isopimpinellin	41 ± 1	IC_50_ > 500 μM	125 ± 13
Toddalolactone	49 ± 5	105 ± 9	220 ± 1
Toddacoumaquinone	46 ± 12	72 ± 5	145 ± 2
Toddanone	46 ± 3	110 ± 2	76 ± 1
Toddaculin	53 ± 9	232 ± 10	143 ± 4
Toddalenone	40 ± 4	211 ± 29	99 ± 1
Artanin	51 ± 2	98 ± 19	124 ± 10
Fraxinol	18 ± 7	IC_50_ > 500 μM	209 ± 21
Tacrine	0.28 ± 0.04	Not detected	Not detected
Curcumin	Not detected	5.8 ± 0.3	5.0 ± 0.1

## Abbreviations

AD	Alzheimer’s disease
Aβ	amyloid beta
AChE	acetylcholinesterase
ACh	acetylcholine
CAS	catalytic anionic site
PAS	peripheral anionic site
APP	amyloid precursor protein
MTDLs	multi-target directed ligands
ThT	Thioflavin T
SAR	the structure–activity relationship
H_2_O_2_	hydrogen peroxide
MTT	3-(4,5-dimethyl-2-thiazolyl)-2,5-diphenyl-2H-tetrazolium bromide
DTNB	5,5′-dithiobis(2-nitrobenzoic acid)
DMSO	dimethyl sulfoxide
